# Trend dynamics of gout prevalence among the Chinese population, 1990-2019: A joinpoint and age-period-cohort analysis

**DOI:** 10.3389/fpubh.2022.1008598

**Published:** 2022-10-12

**Authors:** Bowen Zhu, Yimei Wang, Weiran Zhou, Shi Jin, Ziyan Shen, Han Zhang, Xiaoyan Zhang, Xiaoqiang Ding, Yang Li

**Affiliations:** ^1^Department of Nephrology, Zhongshan Hospital, Fudan University, Shanghai, China; ^2^Shanghai Medical Center of Kidney, Shanghai, China; ^3^Shanghai Key Laboratory of Kidney and Blood Purification, Shanghai, China

**Keywords:** gout, hyperuricemia, epidemiology, joinpoint regression, age-period-cohort analysis, ARIMA model

## Abstract

**Background:**

The burden of gout is increasing worldwide, which places a heavy burden on society and healthcare systems. This study investigates the independent effects of age, period, and cohort on the gout prevalence from 1990 to 2019 in China, compares these effects by gender and then predicts the future burden of gout over the next decade.

**Methods:**

The data were obtained from the Global Burden of Disease (GBD) study in 2019. Joinpoint regression model was employed to calculate the annual percentage change (APC) in gout prevalence, and the age-period-cohort analysis was utilized to estimate the independent effects of age, period, and cohort. ARIMA model was extended to predict the gout epidemic in 2020–2029.

**Results:**

In 2019, there were 16.2 million cases of gout in China, with an age-standardized prevalence rate (ASPR) of 12.3‰ and 3.9‰ in men and women, respectively. During 1990–2019, the ASPR of gout was increasing significantly, with an average APC of 0.9%. The periods of 2014–2017 and 2001–2005 were “joinpoint” for men and women (APC: 6.3 and 5.6%). The age-period-cohort analyses revealed that the relative risk (RR) of developing gout increased with age, peaking at 70–74 years in men (RR_age(70−74)_ = 162.9) and 75–79 years in women (RR_age(75−79)_=142.3). The period effect trended upward, with a more rapid increase in women (RR_period(2019)_ = 2.31) than men (RR_period(2019)_ = 2.23). The cohort effect generally peaked in the earlier cohort born in 1905–1909 for both sexes. Gout prevalence showed a strong positive correlation with the consumption of meat and aquatic products (r_meat_ = 0.966, r_aquaticproducts_ = 0.953). Within 2029, the ASPR of gout was projected to be 11.7‰ and 4.0‰ in men and women, respectively.

**Conclusion:**

The prevalence of gout is increasing at an alarming rate in China; thus, it is necessary to provide targeted health education, regular screening, and accessible urate-lowering therapy healthcare to prevent and protect against gout in China, particularly in older women.

## Introduction

Gout is a common metabolic disease, that results from purine metabolism disorders and/or decreased uric acid excretion (1, 2). It is manifested as hyperuricemia, acute gouty arthritis, chronic gouty arthropathy, renal functional impairment, urolithiasis and obstructive uropathy (3). There have been significant changes in people's lifestyles and eating habits recently with the rapid development of the worldwide economy. It resulted in the increased prevalence of gout and hyperuricemia, which places a heavy burden on society and healthcare systems. According to the 2017 global burden of disease (GBD) study, the prevalence of gout worldwide was 7.9‰ and 2.5‰ in men and women, respectively (4). The US National Health and Nutrition Examination Survey reported that the prevalence of gout increased from 2.9% in 1988–1994 to 3.9% in 2007–2008 (5). In the UK, the gout prevalence was 24.9‰ in 2012, with a 64% increase from 1997 to 2012 (6). In Korea, the gout prevalence increased from 3.5‰ in 2007 to 7.6‰ in 2015 (7). However, there is no national epidemiological survey on the prevalence of gout in China, but meta-analyses estimated that the pooled prevalence of gout is 1.1% (8). The risk of gout increases with age; thus, it is more common in aging populations (9). Previous studies revealed that gout and hyperuricemia were associated with genetic background, a high-purine diet (particularly meat and seafood) and consumption of alcohol and sugar-sweetened beverages (10–13). Moreover, early-life exposure to famine was more likely to develop hyperuricemia in adulthood (14, 15). These age, period, and cohort effects together contributed to the high burden of gout. However, to date, no comprehensive study has explored the longitudinal trends of gout from age, period, and cohort dimensions. Age-period-cohort analysis can estimate these effects on disease, especially in the context of complex historical events and environmental factors (16). Therefore, this study aimed to investigate the independent effects of age, period, and cohort on gout from 1990 to 2019 in China, compare these effects by gender using the GBD 2019 data and then predict the future prevalence of gout over the next decade. The study findings will provide a reliable epidemiology basis for further gout prevention, facilitate adequate healthcare resource planning, and avoid disability in the elderly.

## Methods

### Data sources

Data on gout prevalence during 1990–2019 were retrieved from the world health organization (WHO) GBD estimates (https://ghdx.healthdata.org/). The latest GBD study in 2019 covered 204 countries and territories, providing a standardized and comprehensive estimation of 369 diseases and injuries and 87 risk factors (17, 18). It estimated incidence, prevalence, mortality, years lived with disability (YLDs), years of life lost (YLLs), and disability-adjusted life-years (DALYs) for different age groups, genders, geographical units, time periods, and cause levels. A total of 86,249 sources were used in the GBD estimation process, including censuses, household surveys, civil registration, vital statistics, disease registries, health service use, air pollution monitors, satellite imaging, disease notifications, and other sources (17). In China, GBD data were from the national population consensus, disease surveillance points, maternal and child health surveillance system, chronic disease and risk factor surveillance, as well as surveys (19). The data reliability and population representativeness have been officially recognized, and multiple studies using China GBD data have been published in the top research journals (20, 21). Gout is defined as the presence of characteristic urate crystals in the joint fluid, and/or a tophus proved to contain urate crystals by chemical or polarized light microscopic means, and the presence of six of the twelve gout clinical, laboratory, and X-ray phenomena (22). In this study, we filtered the disease as “gout (B.11.5),” location as “China,” metrics as “prevalence” and “DALY,” and set other options to select all. The crude prevalence rate (CPR) refers to the actual prevalence of all-age populations, and the age-standardized prevalence rate (ASPR) was based on GBD 2019 global age-standard population. Gout prevalence was expressed as a per thousand (‰). Since gout is a non-fatal disease, the estimates of mortality and YLLs were inaccessible in GBD 2019 database. We used the DALYs to measure the healthy life lost due to gout. Based on 10,000 iterations, the uncertainty interval (UI) is defined by the 2.5th and 97.5th draw values, representing the 2.5th and 97.5th percentiles. The sociodemographic index (SDI) of China was also extracted from GBD website (https://vizhub.healthdata.org/gbd-results/). SDI summarizes the level of national development, which is closely related to the resident's health status. The value range of SDI is (0, 1), with higher scores representing higher per capita income and education levels, but lower fertility rates. Moreover, we extracted the national per capita consumption of major foods, including grains, fresh vegetables, vegetable oil, meat (pork, beef, mutton, poultry) and aquatic products, from China Statistical Yearbook 1990–2019 (http://www.stats.gov.cn/tjsj/ndsj/).

### Statistical analysis

Joinpoint analysis was applied to estimate the trends of gout prevalence from 1990 to 2019. As proposed by Kim in 2000 (23), it can divide the longitudinal variations into different segments by piecewise regression and identify the segment trends with statistical significance. Regression fitting was performed on the natural logarithm of the prevalence and mortality rate in different segments, and then the annual percentage change (APC) and its 95% confidence interval (CI) were calculated for each period. The global trend was described by average annual percent change (AAPC). APC and AAPC were considered statistically significant by non-overlapping 95% CI and *p* < 0.05 compared to the null hypothesis of having no variation.

An age-period-cohort model was applied to assess the impact of age, period, and cohort effects on health outcomes (24). The age effect refers to the differences in gout prevalence across age groups caused by aging-related factors. The period effect refers to the influence of human factors on gout prevalence, such as diagnosis development. The cohort effect refers to the change in gout prevalence due to different exposures to risk factors among people of different birth years. Age and period were first divided into 5-year continuous intervals from 15–19 to 85–89, and from 1994–1999 to 2014–2019, respectively. Twenty birth cohorts were summarized from 1905–1909 to 2000–2004. The intrinsic estimator (IE) method was integrated into the age–period–cohort model to estimate the net effects for three dimensions (25). The relative risk (RR) and 95% CI were then calculated based on the estimated coefficients to quantify the effects of age, period, and cohort on gout prevalence. The first groups of 15–19 years, 1994–1999 period, and 1905–1909 birth cohort were defined as the reference groups. Moreover, we applied the Pearson correlation coefficient (r) to evaluate the linear association of gout prevalence and SDI and the national per capita consumption of major foods.

The autoregressive integrated moving average (ARIMA) model was applied to predict future trends of gout prevalence over the next decade. The model expression is ARMIA (p, d, q), where p is the autoregressive order, d is the number of differences, and q is the moving average order (26). The difference method was employed to transform the non–stationary data into stationary data. The autocorrelation function (ACF) and the partial autocorrelation function (PACF) were then plotted to check the stationary of the sequence after differencing, and *auto.arima()* was used to establish the optimal model according to the Akaike information criterion (AIC) value. The *auto.arima()* function is suitable for different ARIMA models of univariate time series data, searches the models according to the provided constraint order, and determines the optimal model (27, 28). The normality of model residuals was tested through QQ plots, ACF and PACF plots. The Ljung–Box test for white noise was used to test whether the residuals have serial correlations. The predictive capacity of ARIMA models was estimated by using mean error (ME), root mean squared error (RMSE), mean absolute error (MAE), mean percentage error (MPE), mean absolute percentage error (MAPE), and mean absolute scaled error (MASE).

The joinpoint analysis was run in the joinpoint regression 4.9 software (Statistical Research and Applications Branch, National Cancer Institute, USA). The age–period–cohort model was established in the Stata 14.0 software (StataCorp LP, TX, UA). The ARIMA analysis and plot drawing were mainly conducted in the R 4.1 software (R core team) using the packages of “forecast,” “tseries” and “ggplot2.” A *p*–value of < 0.05 was considered statistically significant.

## Results

### Description analysis of gout prevalence in China

**Figure 1:**
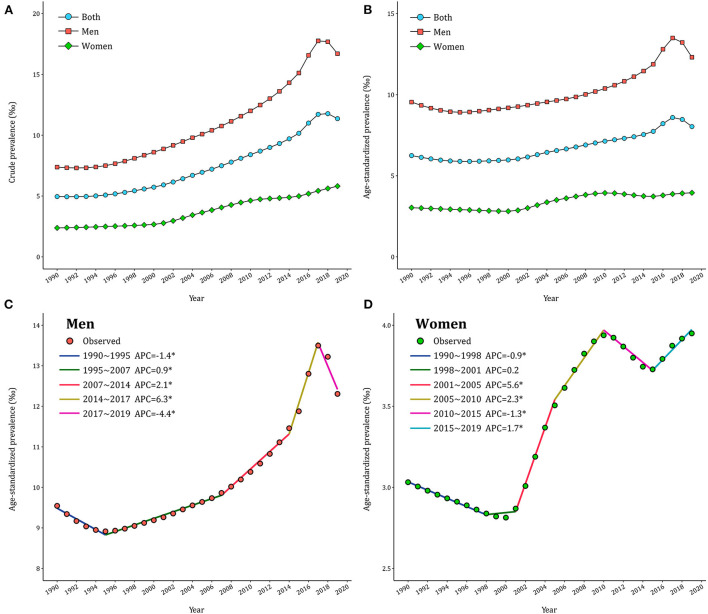
Trends of gout prevalence in China during 1990–2019. **(A)** Crude prevalence of gout. **(B)** Age–standardized prevalence of gout. **(C,D)** APCs of the age–standardized prevalence of gout for men and women.

In 2019, there were 16.2 million (95% UI: 12.8–20.4) cases of gout in China, with men and women patients accounting for 12.1 million (95% UI: 9.6–15.2) and 4.1 million (95% UI: 3.2–5.2), respectively. The CPR and ASPR trends from 1990 to 2017 for gout among Chinese adults were presented in Figure 1 and Supplementary Table 1. In general, the CPR and ASPR of gout were both higher in men than in women. CPR in men increased from 7.38‰ in 1990 to 17.76‰ in 2017 and then decreased to 16.70‰ in 2019. CPR in women gradually increased from 2.38‰ to 5.81‰ during the same period in China. The trends of ASPR by gender were similar to that of CPR but with mild fluctuation. Over the past thirty years, the crude DALYs of gout increased by 124% in men and 141% in women, but the age–adjusted DALYs showed a less marked increase (Supplementary Figure 1).

### Temporal trends of gout prevalence in China

The joinpoint models were applied to divide the temporal trends of gout prevalence into several segments and estimate the APCs by gender. As displayed in Figure 1C, ASPR of gout in men declined first (*APC*_1990−1995_ = −1.4%), then significantly increased (*APC*_1995−2007_ = 0.9% and *APC*_2007−2014_ = 2.1%), peaking in 2017 (*APC*_2014−2017_ = 6.3%) and then decreased thereafter (*APC*_2017−2019_ = −4.4%). By contrast, ASPR underwent three significant increases in women (*APC*_2001−2005_=5.6%, *APC*_2005−2010_ = 2.3% and *APC*_2015−2019_ = 1.7%) and two significant declines (*APC*_1990−1998_ = −0.9% and *APC*_2010−2015_ = −1.3% in 2010–2015) in Figure 1D. Over the entire study period, AAPC was 0.9% (95% CI: 0.8%−1.1%) in men and 0.9% (95% CI: 0.8%−1.0%) in women. Further analyses in CPR of gout across gender exhibited similar patterns (Supplementary Table 2).

### Age, period, and cohort trends of gout prevalence

**Figure 2:**
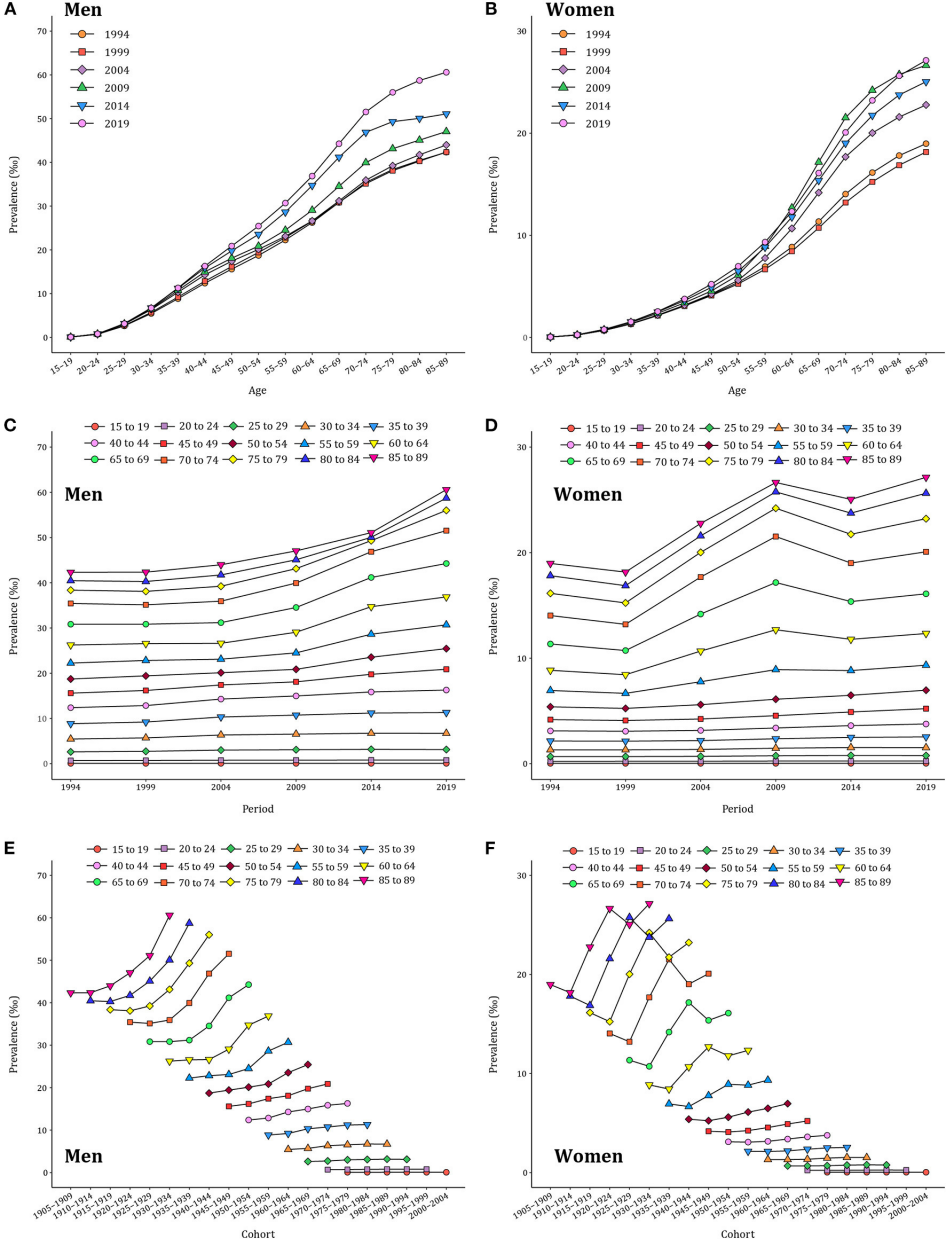
Long–term trends of age–specific, period–based, and cohort–based variation of gout prevalence in China during 1990–2019. **(A,B)** Age–specific prevalence of gout for men and women. **(C,D)** Period–based prevalence of gout for men and women. **(E,F**) Cohort–based prevalence of gout for men and women.

The age–specific gout prevalence was approximated by a linear distribution in different periods (Figures 2A,B). It accelerated with age, reaching a peak in the group aged 85–89 years, and this age pattern was consistent across both men and women. The period variations of gout prevalence in men were relatively stable in the younger groups and trended upward over the period in the 85–89 age group (Figure 2C). In women, by contrast, a period–specific trend with three inflection points became apparent after the groups aged 60–64 years (Figure 2D). The birth cohort of each age group revealed that the gout prevalence in the early period was lower than that in the later period (Figures 2E,F). For men in the 85–89 age group, the gout prevalence increased with the birth cohorts. For women in the 85–89 age group, the gout prevalence increased first and then decreased with the birth cohort. Starting in the 35–39 age group, such trends were leveling–off.

### The age, period, and cohort effects on gout prevalence

**Figure 3:**
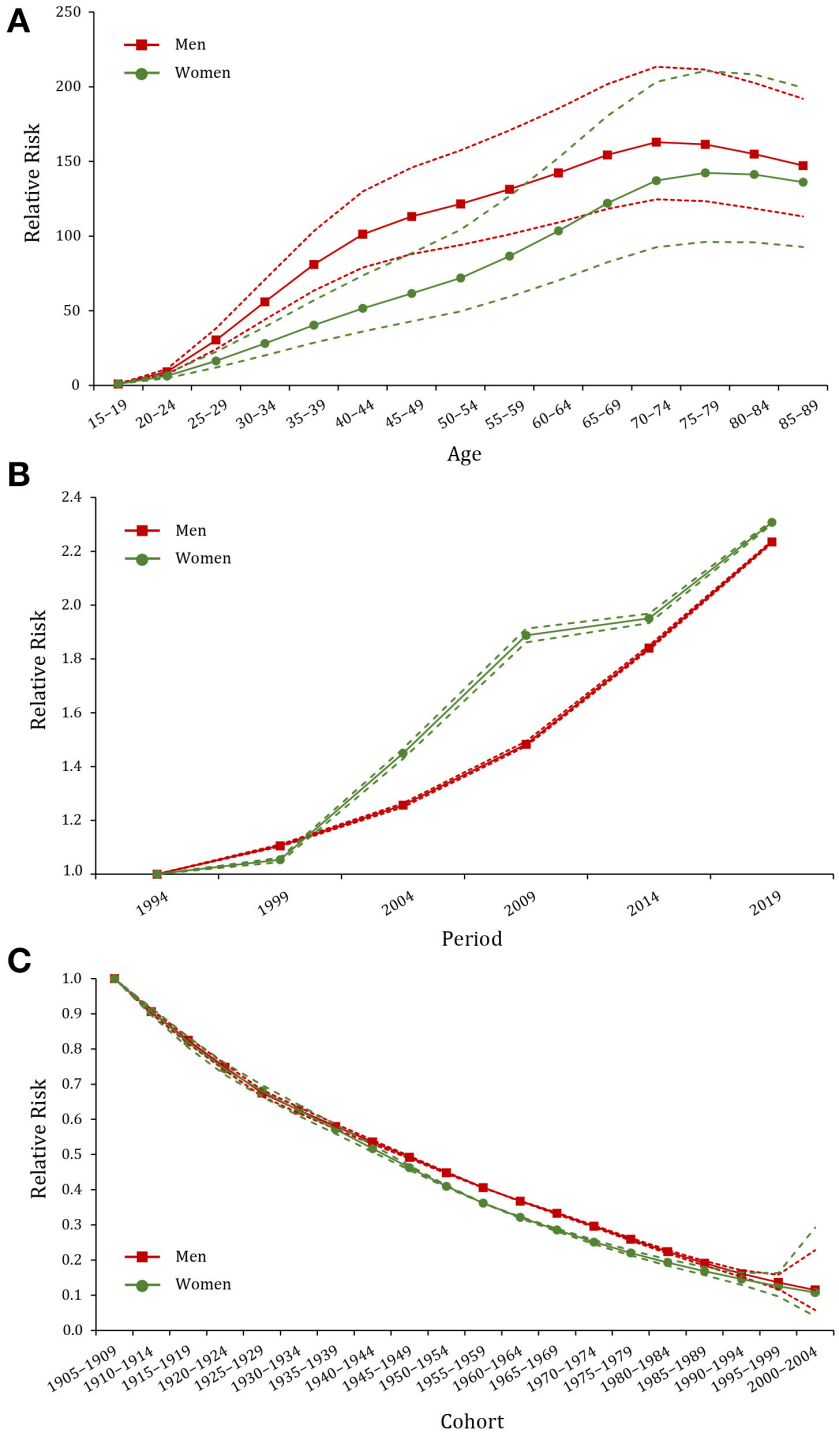
Age, period, and cohort effects on the prevalence of gout in China during 1990–2019. **(A–C)** Age, period, and cohort effects, respectively; the red dot line represents the 95% confidence interval (CI) for men, and the green dot line represents the 95% CI for women.

RRs of age, period, and cohort effects of gout prevalence for both sexes were presented in Figure 3 and Supplementary Tables 3–5. After controlling for period and cohort factors, age was significantly associated with gout prevalence, with the risk increasing with advancing age and then remaining stable thereafter. Regarding the reference group of 15–19 years, RR values peaked at 70–74 years in men (RR_age(70−74)_ = 162.9, 95% CI: 124.6–213.4) and 75–79 years in women (RR_age(75−79)_ = 142.3, 95% CI: 96.1–210.6). The period effect of gout prevalence for both men and women trended upward between 1994 and 2019, with a faster increase in women (RR_period(2019)_ = 2.31, 95% CI: 2.30–2.31) than men (RR_period(2019)_ = 2.23, 95% CI: 2.23–2.24). The cohort effect of gout prevalence showed a significant downward trend and was slightly lower in men than women. The early birth cohort had a greater impact on the risk of gout (RR_cohort(1910−1914)_ = 0.91, 95% CI: 0.90–0.92), which continued to decline in the recent birth cohorts (RR _cohort(2000−2004)_ = 0.12, 95% CI: 0.05–0.27).

### Gout burden and its associated factors

**Figure 4:**
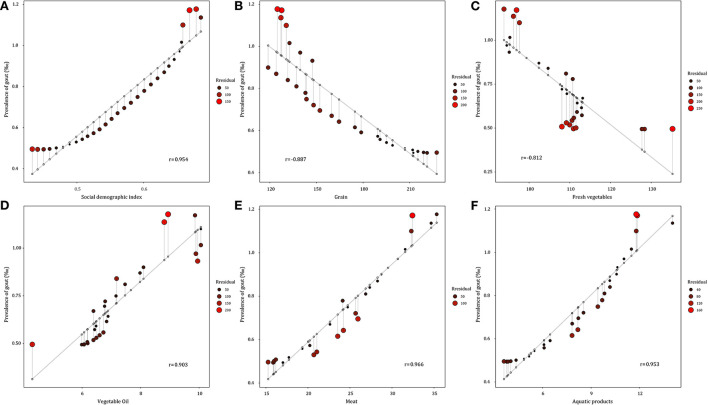
Correlation between gout prevalence and social demographic index and major food consumption from 1990 to 2019 in China. **(A)** Social demographic index. **(B)** Grain consumption. **(C)** Fresh vegetable consumption. **(D)** Vegetable oil consumption. **(E)** Meat consumption. **(F)** Aquatic products consumption.

High body–mass index (BMI) and kidney dysfunction were identified as major risk factors for gout, contributing to 22.5% and 9.1% of gout DALYs, in 2019. As shown in Figure 4, the gout prevalence was positively correlated with SDI, meat, aquatic products, and oil consumption, with a correlation coefficient ranging from 0.903 to 0.966. In contrast, the gout prevalence showed a negative correlation with the consumption of grain and vegetables (r_grain_ = −0.887, r_vegatables_ = −0.812).

### Predicted trends of gout prevalence in 2020–2029

**Figure 5:**
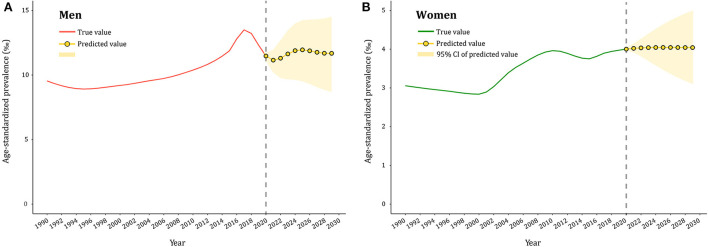
Predicted trends of gout prevalence in China over 10 years (2020–2029). **(A,B)** the red and green lines represent the true trend of age–standardized prevalence of gout during 1990–2019 for men and women; the yellow lines represent the predicted trend and the light–yellow shaded regions represent the 95% confidence interval of predicted values; the gray dot vertical line split data into true value (1990–2019) and predicted value [2020–2029)].

The gout prevalence data from 1990 to 2019 was then applied to quantitatively predict future trends over the next decade in ARIMA models. As presented in Supplementary Figures 2A–C, 3, the longitudinal ASPRs of gout were non–stationary; therefore, first–order differencing was performed to stabilize the variance of the series (Supplementary Figures 2D,F, 4). The differential time series were further verified as non–random series through the white noise test (Supplementary Table 6). Filtered by the *auto.arima()* function, the optimized parameters for ARIMA model were chosen to be (2,1,1) for both men and women, with AICs of 250.78 and 130.02, respectively. Q–Q plots, ACF and PACF plots revealed that the residual error was normally distributed (Supplementary Figure 5). The Ljung–Box test confirmed that ARIMA models were robust and the residuals were white noise (χ^2^ = 0.040/0.004, *p* = 0.842/0.949). The calibration plots suggested that the true value agreed well with the predicted value (Supplementary Figure 6). ARIMA (2,1,1) models were then used to predict ASPR of gout from 2020 to 2029 by gender, as displayed in Figure 5 and Supplementary Figure 7. ASPR in men is expected to increase from 11.47‰ in 2020 to 11.95‰ in 2025, and then decrease to 11.67‰ in 2029, whereas ASPR in women will remain stable in the next decade, ranging from 3.97 to 4.02‰. The predictive capacity of ARIMA models was listed in Supplementary Table 7.

## Discussion

The present study analyzed the temporal trends in gout prevalence in China from 1990 to 2019 and found that it increased significantly over the past three decades. In 2019, there were 16.2 million (95% UI: 12.8–20.4) cases of gout in China, with an ASPR of 12.31‰ (men) and 3.95‰ (women). It was higher than the global estimates (10.31/3.03‰) and other Asian countries (Japan: 11.91/2.72‰, South Korea: 11.60/2.62‰) (29). The increased gout burden is correlated with lifestyle changes, increased life expectancy, and a high prevalence of obesity and other comorbidities (30, 31). Asymptomatic hyperuricemia is a “subclinical or hidden” stage of gout, and its early prevention and treatment are usually neglected (32). Our previous meta–analysis estimated the prevalence of hyperuricemia in China to be 16.4% (33). When uric acid crystals involved musculoskeletal structures, gout can affect patients' ability to perform normal self–care activities, recreational and social activities and work (34, 35). Furthermore, gout–related disability is an underestimated and understudied problem, and there were almost 1.3 million YLDs due to gout in 2017 (36), incurring substantially greater direct and indirect costs ($172 to $6179 per capita) (37). Gout is more prevalent in men than women. This sex disparity was due to estrogen and progesterone promoting uric acid excretion (38), as well as men's greater exposure to risk factors such as smoking, alcohol consumption and obesity (12, 39). Joinpoint analysis revealed that the gout ASPR increased in men and women initially from 2000 to 2010. Subsequently, the prevalence in men began to decline but continued to rise in women and peaked in 2019. The National Health and Nutrition Examination Survey (NHANES) in the United States also showed that the annual increase of gout was only observed in women (from 2.0% in 2007 to 2.7% in 2015) rather than men (40). This may be because women's exposure to unhealthy diets and lifestyles is starting to approach that of men. Although effective and low–cost urate–lowering therapy (ULT) has been available for decades, gender inequities exist in gout management (41). Federica et al. found that women were less likely to participate in clinical trials of serum uric acid lowering drugs (42). Language barriers, disparities in socioeconomic position and cultural practices that limited their participation in ULT need to be acknowledged. In addition, physician attitudes, communication styles and perceptions of patient distrust have been also shown to have a negative impact. Therefore, we should pay more attention to the prevention and control of gout in women, the ever–growing group of gout patients.

Due to the intricate interaction among age, period, and cohort factors, we applied the age–time–cohort model and IE algorithm to quantify their net effects on gout prevalence. It was observed that the age effect increased from the youngest age group to the 70–74 age group and subsequently remained stable. Aging can cause renal morphologic and pathophysiologic dysfunction, resulting in impaired uric acid excretion and elevated serum levels. Moreover, elderly people usually suffer from multiple diseases, such as hypertension, diabetes and cardiovascular diseases, which are involved in gout development (43). Stratified by gender, RRs of the age effect increased faster in women than men in the 55 ~ age group, suggesting that the age effect may be intensified in older women. The decline in estrogen function is the main cause of gout for postmenopausal women. The hyperuricemia and inflammation of gout put women, but not men, at higher risk for osteoporotic fractures (44). Previous studies proved that postmenopausal hormone therapy modestly reduces the risk of gout (45).

In addition, the period effect on gout prevalence remarkably increased in China, which may be explained by the changes in dietary patterns and the increasing obese population. Over the past decades, consumption of energy/fat–dense foods like meat, seafood, alcohol and sugar beverages has increased significantly, with dietary patterns switching from a predominantly plant–based diet to a Western–style diet high in fat and animal–based foods (46). These dietary factors can increase urate concentrations, hence the risk of gout occurrence and progression (47). We also found that gout prevalence showed a strong positive correlation with the consumption of meat and aquatic products (r_meat_ = 0.966, r_aquaticproducts_ = 0.953). A prospective cohort study demonstrated that a DASH–style diet (high intake of fruits, vegetables, nuts and legumes, low–fat dairy and whole grains, and low intake of sodium, sweetened beverages, red and processed meats) could reduce uric acid levels in individuals with hyperuricemia, thereby reducing the risk of gout (48, 49). Obesity, especially abdominal obesity, is also closely linked to gout. According to the China Chronic Disease and Risk Factors Surveillance, the BMI levels rose from 22.7 kg/m^2^ in 2004 to 24.4 kg/m^2^ in 2018 and obesity prevalence from 3.1% to 8.1% (50). Available evidence supports weight loss in overweight/obese gout patients to reduce serum uric acid and gout (51). This study determined high BMI as the major risk factor for gout, contributing to 22.5% of the gout DALYs in 2019. The increasing prevalence of gout makes it necessary to strengthen preventive actions, such as promoting a rational diet, appropriate exercise, and controlling alcohol and tobacco consumption.

The cohort effect represents early socioeconomic, behavioral, and environmental factors on the risk of gout. RRs of the cohort effect initially peaked in the earliest birth cohort (1905–1909) and exhibited a downward trend until the most recent birth cohort (2000–2004). This decline was the result of China's socioeconomic development and medical advancement. However, the gout prevalence still increased over time in the cohort from 1905–1090 to 1949–1950, mainly because of social upheaval, poor nutrition intake, and healthcare conditions before establishing the People's Republic of China in 1949. Since the reform and opening–up policy in China, the prevalence has become stable after the 35–39 age group, whose earliest birth cohort was recorded in 1955–1959. In addition, health awareness has improved among the younger generations, and they devote more attention to chronic disease prevention.

Predicting the gout epidemic is useful for informing the disease burden and assisting in decision–making for health resource allocation. In 2029, ASPR of gout is expected to rise to 11.7‰ and 4.0‰ in men and women, respectively. However, gout is often misdiagnosed as a sprain or infection at the first presentation, or the diagnosis is delayed in many cases (52). Furthermore, the proportion of gout patients receiving ULT was also low, with an overall adherence rate of 47% (53). Studies in China revealed that the rate of ≥80% ULT adherence ranged from 9.6% to 21.9% (54, 55). Absent or delayed use of ULT increases the risk of gout attacks and joint inflammation and destruction, as well as the long–term deleterious effects on the cardiovascular and renal systems. Accordingly, a comprehensive strategy, including risk factor prevention, regular monitoring of uric acid levels, and the popularity of ULT, is necessary to slow the gout epidemic and achieve better health outcomes for gout patients.

This study has several limitations. First, the gout data were extracted from GBD 2019, which had varied data sources including surveillance system data and individual–level survey data, implying that the selection bias could affect the certainty of gout burden estimates. Second, the ASPR of gout was calculated using the GBD standard population rather than the Chinese population. While it is favorable for horizontal comparison with other countries, it might underestimate the actual prevalence due to the larger proportion of the aging population in China. Third, due to the unavailability of provincial data, this study did not have a geographic description of the burden of gout. It prevents us from validating the ARIMA predictions at the provincial level. Our previous meta–analysis found that hyperuricemia prevalence was higher in southern and southwestern China, possibly attributed to regional eating habits (seafood and hot pot) (33). Lastly, the age–period–cohort model was analyzed at the population level, so that it may be subject to ecological fallacy.

## Conclusion

The prevalence of gout in Chinese men and women increased at an alarming rate from 1990 to 2019, with age being the critical factor affecting the gout epidemic. China's population growth and aging also amplified this effect, and there was a persistent impact of an unhealthy diet and obesity on gout prevalence in China. Therefore, it is necessary to provide targeted health education, regular screening and accessible ULT healthcare to reduce the gout disease burden. Moreover, preventing and protecting against gout should be reinforced for older women.

## Data availability statement

Publicly available datasets were analyzed in this study. This data can be found here the Global Burden of Disease study 2019 is an open–access resource, data are available at https://vizhub.healthdata.org/gbd-results/.

## Author contributions

YL and XD contributed to the conception and design of the work. BZ, YW, and WZ contributed to the acquisition, analysis, and interpretation of data for the work. SJ, ZS, and HZ participated in data management. BZ, YW, and YL drafted the manuscript. XZ, XD, and YL critically revised the manuscript. All authors gave final approval and agree to be accountable for all aspects of work ensuring integrity and accuracy.

## Funding

This analytic study was sponsored by the National Natural Science Foundation of China (82103911), Natural Science Foundation of Shanghai (21ZR1412400), Shanghai Municipal Key Clinical Specialty (shslczdzk02501), and Shanghai Key Laboratory of Kidney and Blood Purification (20DZ2271600).

## Conflict of interest

The authors declare that the research was conducted in the absence of any commercial or financial relationships that could be construed as a potential conflict of interest.

## Publisher's note

All claims expressed in this article are solely those of the authors and do not necessarily represent those of their affiliated organizations, or those of the publisher, the editors and the reviewers. Any product that may be evaluated in this article, or claim that may be made by its manufacturer, is not guaranteed or endorsed by the publisher.

## Abbreviations

AAPC: average annual percent change
ACF: autocorrelation function
AIC: Akaike information criterion
APC: annual percentage change
ARIMA: autoregressive integrated moving average
ASPR: age-standardized prevalence rate
BMI: body-mass index
CPR: crude prevalence rate
CI: confidence interval
DALYs: disability-adjusted life-years
GBD: global burden of disease
IE: intrinsic estimator
PACF: partial autocorrelation function
RR: relative risk
SDI: sociodemographic index
UI: uncertainty interval
ULT: urate-lowering therapy
WHO: world health organization
YLDs: years lived with disability
YLLs: years of life lost.

## References

[1] Dalbeth N Gosling AL Gaffo A Abhishek A. Gout. Lancet (London, England). (2021) 397:1843–55. 10.1016/S0140-6736(21)00569-9 33798500

[2] Terkeltaub R Bushinsky DA Becker MA. Recent developments in our understanding of the renal basis of hyperuricemia and the development of novel antihyperuricemic therapeutics. Arthritis Res Ther. (2006) 8 (Suppl. 1):S4. 10.1186/ar1909 16820043 PMC3226109

[3] Terkeltaub RA. Clinical practice. Gout New Eng J Med. (2003) 349:1647–55. 10.1056/NEJMcp030733 14573737

[4] Xia Y Wu Q Wang H Zhang S Jiang Y Gong T. Global, regional and national burden of gout, 1990-2017: a systematic analysis of the Global Burden of Disease Study. Rheumatology (Oxford, England). (2020) 59:1529–38. 10.1093/rheumatology/kez476 31624843

[5] Zhu Y Pandya BJ Choi HK. Prevalence of gout and hyperuricemia in the US general population: the National Health and Nutrition Examination Survey 2007-2008. Arthritis Rheum. (2011) 63:3136–41. 10.1002/art.30520 21800283

[6] Kuo CF Grainge MJ Mallen C Zhang W Doherty M. Rising burden of gout in the UK but continuing suboptimal management: a nationwide population study. Ann Rheum Dis. (2015) 74:661–7. 10.1136/annrheumdis-2013-204463 24431399 PMC4392307

[7] Kim JW Kwak SG Lee H Kim SK Choe JY Park SH. Prevalence and incidence of gout in Korea: data from the national health claims database 2007-2015. Rheumatol Int. (2017) 37:1499–506. 10.1007/s00296-017-3768-4 28676911

[8] Liu R Han C Wu D Xia X Gu J Guan H. Prevalence of hyperuricemia and gout in Mainland China from 2000 to 2014: a systematic review and meta-analysis. Biomed Res Int. (2015) 2015:762820. 10.1155/2015/762820 26640795 PMC4657091

[9] Dehlin M Jacobsson L Roddy E. Global epidemiology of gout: prevalence, incidence, treatment patterns and risk factors. Nature Rev Rheumatol. (2020) 16:380–90. 10.1038/s41584-020-0441-1 32541923

[10] Major TJ Dalbeth N Stahl EA Merriman TR. An update on the genetics of hyperuricaemia and gout. Nature Rev Rheumatol. (2018) 14:341–53. 10.1038/s41584-018-0004-x 29740155

[11] Choi HK Atkinson K Karlson EW Willett W Curhan G. Purine-rich foods, dairy and protein intake, and the risk of gout in men. N Engl J Med. (2004) 350:1093–103. 10.1056/NEJMoa035700 15014182

[12] Zhu B Li Y Shi Y Song N Fang Y Ding X. Long-term drinking behavior change patterns and its association with hyperuricemia in chinese adults: evidence from China Health and Nutrition Survey. BMC Public Health. (2022) 22:1230. 10.1186/s12889-022-13637-4 35725435 PMC9210654

[13] Ebrahimpour-Koujan S Saneei P Larijani B Esmaillzadeh A. Consumption of sugar sweetened beverages and dietary fructose in relation to risk of gout and hyperuricemia: a systematic review and meta-analysis. Crit Rev Food Sci Nutr. (2020) 60:1–10. 10.1080/10408398.2018.1503155 30277800

[14] Wang Y Weng P Wan H Zhang W Chen C Chen Y. Economic status moderates the association between early-life famine exposure and hyperuricemia in adulthood. J Clin Endocrinol Metab. (2020) 105:dgaa523. 10.1210/clinem/dgaa523 32789437

[15] Zhang W Luan R. Early-life exposure to the Chinese famine of 1959-61 and risk of Hyperuricemia: results from the China health and retirement longitudinal study. BMC Public Health. (2020) 20:15. 10.1186/s12889-019-8017-1 31906901 PMC6945412

[16] SE F WM M.. Identification and estimation of age-period-cohort models in the analysis of discrete archival data. Sociological Methodol. (1979) 10:1–67. 10.2307/270764

[17] GBD 2019 Diseases and Injuries Collaborators. Global burden of 369 diseases and injuries in 204 countries and territories, 1990-2019: a systematic analysis for the Global Burden of Disease Study 2019. Lancet (London, England). (2020) 396:1204-22. 10.1016/s0140-6736(20)30925-9 33069326 PMC7567026

[18] GBD 2019 Viewpoint Collaborators. Five insights from the Global Burden of Disease Study 2019. Lancet (London, England). (2020) 396:1135–59. 10.1016/s0140-6736(20)31404-5 33069324 PMC7116361

[19] Zhou M Wang H Zeng X Yin P Zhu J Chen W. Mortality, morbidity, and risk factors in China and its provinces, 1990-2017: a systematic analysis for the Global Burden of Disease Study 2017. Lancet (London, England). (2019) 394:1145–58. 10.1016/S0140-6736(19)30427-1 31248666 PMC6891889

[20] Liu S Li Y Zeng X Wang H Yin P Wang L. Burden of cardiovascular diseases in China, 1990-2016: findings from the 2016 global burden of disease study. JAMA cardiology. (2019) 4:342–52. 10.1001/jamacardio.2019.0295 30865215 PMC6484795

[21] Wang L Peng W Zhao Z Zhang M Shi Z Song Z. Prevalence and treatment of diabetes in China, 2013-2018. JAMA. (2021) 326:2498–506. 10.1001/jama.2021.22208 34962526 PMC8715349

[22] Wallace SL Robinson H Masi AT Decker JL McCarty DJ Yü TF. Preliminary criteria for the classification of the acute arthritis of primary gout. Arthritis Rheum. (1977) 20:895–900. 10.1002/art.1780200320 856219

[23] Kim HJ Fay MP Feuer EJ Midthune DN. Permutation tests for joinpoint regression with applications to cancer rates. Stat Med. (2000) 19:335. doi:10.1002/(sici)1097-0258(20000215)19:3<335::aid-sim336>3.0.co;2-z 10649300 10.1002/(sici)1097-0258(20000215)19:3<335::aid-sim336>3.0.co;2-z

[24] Rosenberg PS A. new age-period-cohort model for cancer surveillance research. Stat Methods Med Res. (2019) 28:3363–91. 10.1177/0962280218801121 30306829

[25] Luo L. Assessing validity and application scope of the intrinsic estimator approach to the age-period-cohort problem. Demography. (2013) 50:1945–67. 10.1007/s13524-013-0243-z 24072610 PMC5129181

[26] Nguyen HV Naeem MA Wichitaksorn N Pears R A. smart system for short-term price prediction using time series models. ComputersElectrical Engineer. (2019) 76:339–52. 10.1016/j.compeleceng.2019.04.013. 10.1016/j.compeleceng.2019.04.013

[27] Nazari KangavariHShojaeiAHashemi NazariSS. Suicide mortality trends in four provinces of Iran with the highest mortality, from 2006–2016. J Res Health Sci. (2017) 17:e00382.28676594

[28] Zhao X Li C Ding G Heng Y Li A Wang W. The burden of Alzheimer's disease mortality in the United States, 1999-2018. J Alzheimer's Dis. (2021) 82:803–13. 10.3233/JAD-210225 34092643

[29] Institute for Health Metrics and Evaluation. 2019 Global Burden of Disease (GBD). (2020). Available online at: http://ghdx.healthdata.org/gbd-results-tool (accessed March 16, 2022).

[30] Kuo CF Grainge MJ Zhang W Doherty M. Global epidemiology of gout: prevalence, incidence and risk factors. Nature Rev Rheumatol. (2015) 11:649–62. 10.1038/nrrheum.2015.91 26150127

[31] Singh JA Reddy SG Kundukulam J. Risk factors for gout and prevention: a systematic review of the literature. Curr Opin Rheumatol. (2011) 23:192–202. 10.1097/BOR.0b013e3283438e13 21285714 PMC4104583

[32] Littman BH. Asymptomatic hyperuricemia. The case for benign neglect Postgraduate medicine. Postgrad Med. (1985) 77:221–4. 10.1080/00325481.1985.11698994 3991381

[33] Li Y Shen Z Zhu B Zhang H Zhang X Ding X. Demographic, regional and temporal trends of hyperuricemia epidemics in mainland China from 2000 to 2019: a systematic review and meta-analysis. Glob Health Action. (2021) 14:1874652. 10.1080/16549716.2021.1874652 33475474 PMC7833047

[34] Sigurdardottir V Drivelegka P Svärd A Jacobsson LTH Dehlin M. Work disability in gout: a population-based case-control study. Ann Rheum Dis. (2018) 77:399–404. 10.1136/annrheumdis-2017-212063 29170202

[35] Klooster PM Vonkeman HE. van de Laar MA. Disability due to gouty arthritis. Curr Opin Rheumatol. (2012) 24:139–44. 10.1097/BOR.0b013e32834ff59d 22227879

[36] Safiri S Kolahi AA Cross M Carson-Chahhoud K Hoy D Almasi-Hashiani A. Prevalence, incidence, and years lived with disability due to gout and its attributable risk factors for 195 countries and territories 1990-2017: a systematic analysis of the global burden of disease study 2017. Arthritis Rheumatol. (2020) 72:1916–27. 10.1002/art.41404 32755051

[37] Rai SK Burns LC De Vera MA Haji A Giustini D Choi HK. The economic burden of gout: a systematic review. Semin Arthritis Rheum. (2015) 45:75–80. 10.1016/j.semarthrit.2015.02.004 25912932

[38] Sumino H Ichikawa S Kanda T Nakamura T Sakamaki T. Reduction of serum uric acid by hormone replacement therapy in postmenopausal women with hyperuricaemia. Lancet (London, England). (1999) 354:650. 10.1016/S0140-6736(99)92381-4 10466673

[39] Gee Teng G Pan A Yuan JM Koh WP. Cigarette smoking and the risk of incident gout in a prospective cohort study. Arthritis Care Res. (2016) 68:1135–42. 10.1002/acr.22821 26714165 PMC5515666

[40] Chen-Xu M Yokose C Rai SK Pillinger MH Choi HK. Contemporary prevalence of gout and hyperuricemia in the United States and Decadal Trends: the national health and nutrition examination survey, 2007–2016. Arthritis Rheumatol (Hoboken, NJ). (2019) 71:991–9. 10.1002/art.40807 30618180 PMC6536335

[41] Guillén AG Te Karu L Singh JA Dalbeth N. Gender and ethnic inequities in gout burden and management. Rheum Dis Clin North Am. (2020) 46:693–703. 10.1016/j.rdc.2020.07.008 32981646

[42] Fogacci F Borghi C Di Micoli A Degli Esposti D Cicero AFG. Inequalities in enrollment of women and racial minorities in trials testing uric acid lowering drugs. NMCD. (2021) 31:3305–13. 10.1016/j.numecd.2021.09.011 34656384

[43] Singh JA Gaffo A. Gout epidemiology and comorbidities. Semin Arthritis Rheum. (2020) 50:S11–16. 10.1016/j.semarthrit.2020.04.008 32620196

[44] Wang Y Zhou R Zhong W Hu C Lu S Chai Y. Association of gout with osteoporotic fractures. Int Orthop. (2018) 42:2041–7. 10.1007/s00264-018-4033-5 29955945

[45] Hak AE Curhan GC Grodstein F Choi HK. Menopause, postmenopausal hormone use and risk of incident gout. Ann Rheum Dis. (2010) 69:1305–9. 10.1136/ard.2009.109884 19592386 PMC3142742

[46] Bu T Tang D Liu Y Chen D. Trends in dietary patterns and diet-related behaviors in China. Am J Health Behav. (2021) 45:371–83. 10.5993/AJHB.45.2.15 33888196

[47] Major TJ Topless RK Dalbeth N Merriman TR. Evaluation of the diet wide contribution to serum urate levels: meta-analysis of population based cohorts. BMJ (Clinical research ed). (2018) 363:k3951. 10.1136/bmj.k3951 30305269 PMC6174725

[48] Rai SK Fung TT Lu N Keller SF Curhan GC Choi HK. The Dietary Approaches to Stop Hypertension (DASH) diet, Western diet, and risk of gout in men: prospective cohort study. BMJ (Clinical research ed). (2017) 357:j1794. 10.1136/bmj.j1794 28487277 PMC5423545

[49] McCormick N Rai SK Lu N Yokose C Curhan GC Choi HK. Estimation of primary prevention of gout in men through modification of obesity and other key lifestyle factors. JAMA Network Open. (2020) 3:e2027421. 10.1001/jamanetworkopen.2020.27421 33231639 PMC7686865

[50] Wang L Zhou B Zhao Z Yang L Zhang M Jiang Y. Body-mass index and obesity in urban and rural China: findings from consecutive nationally representative surveys during 2004-18. Lancet (London, England). (2021) 398:53–63. 10.1016/S0140-6736(21)00798-4 34217401 PMC7617101

[51] Nielsen SM Bartels EM Henriksen M Wæhrens EE Gudbergsen H Bliddal H. Weight loss for overweight and obese individuals with gout: a systematic review of longitudinal studies. Ann Rheum Dis. (2017) 76:1870–82. 10.1136/annrheumdis-2017-211472 28866649 PMC5705854

[52] Singh JA. Are the days of missed or delayed diagnosis of gout over? Nature Rev Rheumatol. (2019) 15:578–80. 10.1038/s41584-019-0286-7 31406335

[53] Yin R Li L Zhang G Cui Y Zhang L Zhang Q. Rate of adherence to urate-lowering therapy among patients with gout: a systematic review and meta-analysis. BMJ Open. (2018) 8:e017542. 10.1136/bmjopen-2017-017542 29643150 PMC5898304

[54] Sheng F Fang W Zhang B Sha Y Zeng X. Adherence to gout management recommendations of Chinese patients. Medicine. (2017) 96:e8532. 10.1097/MD.0000000000008532 29137059 PMC5690752

[55] Yin R Cao H Fu T Zhang Q Zhang L Li L. The rate of adherence to urate-lowering therapy and associated factors in Chinese gout patients: a cross-sectional study. Rheumatol Int. (2017) 37:1187–94. 10.1007/s00296-017-3746-x 28551724

