# Supplementary material for: Trend dynamics of gout prevalence among the Chinese population, 1990-2019: A joinpoint and age-period-cohort analysis
Source: Front Public Health. 2022 Oct 12;10:1008598. doi: 10.3389/fpubh.2022.1008598 (PMC9602928; doi:10.3389/fpubh.2022.1008598)
Supplement: Supplementary file 1 [file Data_Sheet_1.pdf]

Supplementary Table 1. Gout prevalence in China during 1990-2019

Year	CPR(95%UI)			ASPR(95%UI)		
	Both(‰)	Men(‰)	Women(‰)	Both(‰)	Men(‰)	Women(‰)
1990	4.95(3.92-6.16)	7.38(5.79-9.19)	2.38(1.87-2.99)	6.25(4.98-7.77)	9.54(7.63-11.80)	3.03(2.41-3.80)
1991	4.94(3.91-6.14)	7.33(5.76-9.12)	2.40(1.89-3.01)	6.14(4.90-7.62)	9.34(7.47-11.54)	3.01(2.39-3.77)
1992	4.94(3.92-6.14)	7.31(5.75-9.08)	2.42(1.91-3.03)	6.04(4.83-7.49)	9.17(7.34-11.31)	2.98(2.37-3.72)
1993	4.96(3.93-6.16)	7.33(5.78-9.11)	2.44(1.92-3.05)	5.97(4.77-7.38)	9.04(7.23-11.15)	2.96(2.35-3.70)
1994	5.01(3.97-6.22)	7.39(5.85-9.19)	2.46(1.94-3.08)	5.91(4.73-7.31)	8.95(7.17-11.04)	2.93(2.32-3.67)
1995	5.08(4.03-6.30)	7.50(5.94-9.33)	2.49(1.97-3.11)	5.89(4.71-7.28)	8.92(7.15-11.01)	2.91(2.30-3.65)
1996	5.18(4.11-6.42)	7.66(6.07-9.53)	2.52(2.00-3.15)	5.89(4.71-7.26)	8.93(7.16-11.02)	2.89(2.29-3.62)
1997	5.30(4.20-6.57)	7.87(6.24-9.77)	2.55(2.02-3.19)	5.90(4.72-7.29)	8.98(7.20-11.06)	2.86(2.27-3.59)
1998	5.43(4.31-6.72)	8.10(6.43-10.02)	2.58(2.04-3.24)	5.92(4.74-7.29)	9.05(7.25-11.12)	2.84(2.25-3.55)
1999	5.58(4.43-6.90)	8.36(6.62-10.31)	2.62(2.07-3.29)	5.95(4.77-7.31)	9.12(7.31-11.17)	2.82(2.24-3.53)
2000	5.73(4.55-7.08)	8.61(6.81-10.59)	2.67(2.11-3.35)	5.98(4.79-7.34)	9.19(7.37-11.23)	2.81(2.23-3.53)
2001	5.92(4.70-7.30)	8.87(7.03-10.93)	2.77(2.20-3.48)	6.04(4.84-7.43)	9.26(7.42-11.30)	2.87(2.27-3.60)
2002	6.15(4.89-7.59)	9.17(7.28-11.27)	2.96(2.34-3.72)	6.16(4.94-7.58)	9.36(7.48-11.42)	3.01(2.38-3.76)
2003	6.42(5.11-7.91)	9.48(7.53-11.69)	3.19(2.52-4.02)	6.30(5.06-7.76)	9.46(7.56-11.59)	3.19(2.53-4.00)
2004	6.70(5.33-8.25)	9.80(7.77-12.07)	3.44(2.70-4.32)	6.44(5.18-7.95)	9.56(7.63-11.75)	3.37(2.67-4.22)
2005	6.95(5.54-8.59)	10.09(7.99-12.45)	3.65(2.88-4.59)	6.56(5.26-8.07)	9.64(7.69-11.86)	3.51(2.77-4.39)
2006	7.21(5.74-8.91)	10.40(8.24-12.83)	3.85(3.04-4.83)	6.66(5.34-8.21)	9.73(7.79-11.97)	3.61(2.85-4.53)
2007	7.49(5.98-9.28)	10.76(8.52-13.25)	4.06(3.20-5.09)	6.77(5.43-8.38)	9.86(7.91-12.12)	3.72(2.94-4.68)
2008	7.79(6.22-9.63)	11.14(8.83-13.76)	4.27(3.37-5.36)	6.90(5.52-8.54)	10.02(8.04-12.33)	3.82(3.01-4.81)
2009	8.10(6.46-10.04)	11.56(9.17-14.34)	4.47(3.52-5.63)	7.02(5.61-8.70)	10.19(8.18-12.55)	3.90(3.07-4.90)
2010	8.40(6.69-10.40)	12.00(9.53-14.93)	4.63(3.64-5.83)	7.13(5.71-8.83)	10.38(8.33-12.79)	3.94(3.10-4.95)
2011	8.70(6.92-10.79)	12.48(9.92-15.51)	4.73(3.72-5.95)	7.22(5.78-8.93)	10.59(8.50-13.04)	3.92(3.10-4.93)
2012	9.00(7.16-11.18)	13.01(10.32-16.15)	4.79(3.75-6.03)	7.31(5.84-9.02)	10.83(8.69-13.35)	3.87(3.06-4.86)
2013	9.32(7.42-11.61)	13.61(10.81-16.87)	4.83(3.79-6.08)	7.41(5.92-9.16)	11.11(8.92-13.74)	3.80(3.00-4.77)
2014	9.70(7.72-12.12)	14.32(11.37-17.80)	4.89(3.83-6.19)	7.54(6.03-9.34)	11.46(9.19-14.16)	3.74(2.96-4.70)
2015	10.16(8.08-12.67)	15.12(12.04-18.90)	4.99(3.90-6.33)	7.74(6.18-9.60)	11.88(9.53-14.66)	3.73(2.95-4.69)
2016	11.00(8.73-13.81)	16.57(13.14-20.7)	5.19(4.07-6.57)	8.22(6.55-10.15)	12.80(10.28-15.76)	3.79(3.01-4.76)
2017	11.72(9.27-14.77)	17.76(14.01-22.23)	5.43(4.25-6.85)	8.59(6.82-10.66)	13.50(10.80-16.65)	3.87(3.08-4.86)
2018	11.77(9.34-14.80)	17.69(14.01-22.22)	5.62(4.39-7.13)	8.47(6.75-10.48)	13.22(10.55-16.29)	3.92(3.10-4.92)
2019	11.36(9.00-14.31)	16.70(13.26-20.93)	5.81(4.54-7.40)	8.03(6.42-9.94)	12.31(9.80-15.22)	3.95(3.12-4.97)

Supplementary Table 2. APCs and AAPCs of gout prevalence of gout in China during 1990-2019

Segments	Age-standardized prevalence			Crude prevalence		
	Year	APC (95% CI)	p-value	Year	APC (95% CI)	p-value
**Both**						
Trend 1	1990-1994	-1.4(-1.7~-1.2)	<0.001	1990-1994	0.1(-0.1~0.4)	0.233
Trend 2	1994-2000	0.2(0.0~0.4)	0.022	1994-2000	2.3(2.1~2.5)	<0.001
Trend 3	2000-2009	1.9(1.8~2.0)	<0.001	2000-2009	4.0(3.9~4.1)	<0.001
Trend 4	2009-2014	1.2(1.0~1.5)	<0.001	2009-2014	3.5(3.2~3.7)	<0.001
Trend 5	2014-2017	4.8(4.0~5.6)	<0.001	2014-2017	6.9(6.0~7.7)	<0.001
Trend 6	2017-2019	-3.1(-3.8~-2.3)	<0.001	2017-2019	-1.3(-2.0~-0.6)	0.002
AAPC	1990-2019	0.9(0.8~1.0)	<0.001	1990-2019	2.9(2.8~3.0)	<0.001
**Male**						
Trend 1	1990-1995	-1.4(-1.6~-1.2)	<0.001	1990-1995	0.2(0.0~0.4)	0.062
Trend 2	1995-2007	0.9(0.8~0.9)	<0.001	1995-2008	3.1(3.1~3.2)	<0.001
Trend 3	2007-2014	2.1(1.9~2.2)	<0.001	2008-2014	4.2(4.0~4.4)	<0.001
Trend 4	2014-2017	6.3(5.3~7.2)	<0.001	2014-2017	8.1(7.0~9.1)	<0.001
Trend 5	2017-2019	-4.4(-5.3~-3.5)	<0.001	2017-2019	-2.9(-3.8~-1.9)	<0.001
AAPC	1990-2019	0.9(0.8~1.1)	<0.001	1990-2019	2.9(2.8~3.1)	<0.001
**Female**						
Trend 1	1990-1998	-0.9(-0.9~-0.8)	<0.001	1990-1998	1.0(0.9~1.1)	<0.001
Trend 2	1998-2001	0.2(-0.5~1.0)	0.547	1998-2001	2.4(1.6~3.2)	<0.001
Trend 3	2001-2005	5.6(5.2~6.0)	<0.001	2001-2005	7.4(7.0~7.9)	<0.001
Trend 4	2005-2010	2.3(2.1~2.6)	<0.001	2005-2010	4.9(4.6~5.1)	<0.001
Trend 5	2010-2015	-1.3(-1.5~-1.0)	<0.001	2010-2015	1.3(1.1~1.6)	<0.001
Trend 6	2015-2019	1.7(1.4~1.9)	<0.001	2015-2019	4.0(3.8~4.3)	<0.001
AAPC	1990-2019	0.9(0.8~1.0)	<0.001	1990-2019	3.2(3.0~3.3)	<0.001

APCs, annual percent changes; AAPCs, average annual percent changes.

Supplementary Table 3. Age, period and cohort effects of gout prevalence in China

Factors	Effect coefficient	Standard error	Z value	p-value	95% CI	
					Lower	Upper
Age						
15-19	-4.108	0.171	-24.040	<0.001	-4.443	-3.773
20-24	-2.004	0.058	-34.810	<0.001	-2.117	-1.891
25-29	-0.845	0.038	-22.190	<0.001	-0.920	-0.771
30-34	-0.244	0.032	-7.750	<0.001	-0.306	-0.183
35-39	0.127	0.027	4.620	<0.001	0.073	0.181
40-44	0.353	0.024	14.550	<0.001	0.306	0.401
45-49	0.475	0.022	22.050	<0.001	0.433	0.517
50-54	0.559	0.019	29.520	<0.001	0.522	0.596
55-59	0.658	0.016	39.950	<0.001	0.626	0.690
60-64	0.757	0.014	53.150	<0.001	0.729	0.785
65-69	0.852	0.013	67.990	<0.001	0.827	0.877
70-74	0.915	0.012	78.720	<0.001	0.892	0.937
75-79	0.906	0.012	76.940	<0.001	0.882	0.929
80-84	0.851	0.013	65.990	<0.001	0.826	0.877
85-89	0.749	0.015	49.870	<0.001	0.720	0.779
Period						
1994	-0.373	0.010	-36.050	<0.001	-0.393	-0.352
1999	-0.290	0.008	-36.220	<0.001	-0.305	-0.274
2004	-0.106	0.006	-16.440	<0.001	-0.119	-0.093
2009	0.091	0.006	14.560	<0.001	0.079	0.104
2014	0.247	0.008	32.930	<0.001	0.232	0.262
2019	0.430	0.009	46.270	<0.001	0.412	0.448
Cohort						
1905-1909	0.918	0.031	29.550	<0.001	0.857	0.979
1910-1914	0.823	0.026	31.070	<0.001	0.771	0.875
1915-1919	0.742	0.024	30.760	<0.001	0.695	0.789
1920-1924	0.666	0.023	29.330	<0.001	0.621	0.710
1925-1929	0.575	0.022	26.110	<0.001	0.532	0.618
1930-1934	0.500	0.022	22.910	<0.001	0.457	0.543
1935-1939	0.427	0.023	18.850	<0.001	0.382	0.471
1940-1944	0.340	0.024	14.230	<0.001	0.293	0.387
1945-1949	0.243	0.025	9.520	<0.001	0.193	0.293
1950-1954	0.134	0.027	4.880	<0.001	0.080	0.188
1955-1959	0.032	0.030	1.080	0.278	-0.026	0.090
1960-1964	-0.076	0.032	-2.380	0.017	-0.138	-0.013
1965-1969	-0.183	0.034	-5.340	<0.001	-0.251	-0.116
1970-1974	-0.297	0.037	-8.030	<0.001	-0.370	-0.225
1975-1979	-0.432	0.040	-10.720	<0.001	-0.510	-0.353
1980-1984	-0.576	0.045	-12.870	<0.001	-0.664	-0.489
1985-1989	-0.729	0.052	-13.970	<0.001	-0.831	-0.627
1990-1994	-0.882	0.069	-12.860	<0.001	-1.017	-0.748
1995-1999	-1.029	0.123	-8.390	<0.001	-1.270	-0.789
2000-2004	-1.194	0.435	-2.740	0.006	-2.047	-0.341
Intercept	6.586	0.023	288.730	<0.001	6.542	6.631
Log likelihood	-403.872					
AIC	9.819					
BIC	-187.389					

Supplementary Table 4. Age, period and cohort effects of gout prevalence in China (for men)

Factors	Effect coefficient	Standard error	Z value	p-value	95% CI	
					Lower	Upper
Age						
15-19	-4.218	0.147	-28.680	<0.001	-4.507	-3.930
20-24	-2.012	0.047	-42.490	<0.001	-2.105	-1.920
25-29	-0.804	0.031	-25.760	<0.001	-0.865	-0.743
30-34	-0.194	0.026	-7.480	<0.001	-0.245	-0.143
35-39	0.176	0.023	7.760	<0.001	0.131	0.220
40-44	0.399	0.020	19.900	<0.001	0.360	0.439
45-49	0.511	0.018	28.650	<0.001	0.476	0.546
50-54	0.582	0.016	37.010	<0.001	0.552	0.613
55-59	0.660	0.014	48.030	<0.001	0.633	0.687
60-64	0.740	0.012	61.880	<0.001	0.716	0.763
65-69	0.821	0.011	77.830	<0.001	0.800	0.842
70-74	0.875	0.010	89.640	<0.001	0.856	0.895
75-79	0.866	0.010	88.270	<0.001	0.847	0.885
80-84	0.825	0.011	77.540	<0.001	0.804	0.845
85-89	0.774	0.012	63.180	<0.001	0.750	0.798
Period						
1994	-0.356	0.008	-42.090	<0.001	-0.373	-0.340
1999	-0.256	0.007	-39.200	<0.001	-0.268	-0.243
2004	-0.128	0.005	-23.920	<0.001	-0.139	-0.118
2009	0.038	0.005	7.270	<0.001	0.028	0.049
2014	0.254	0.006	41.250	<0.001	0.242	0.266
2019	0.448	0.008	58.790	<0.001	0.433	0.462
Cohort						
1905-1909	0.941	0.026	36.580	<0.001	0.891	0.992
1910-1914	0.843	0.022	37.930	<0.001	0.800	0.887
1915-1919	0.748	0.020	36.510	<0.001	0.708	0.788
1920-1924	0.651	0.019	33.490	<0.001	0.613	0.689
1925-1929	0.548	0.019	28.920	<0.001	0.511	0.585
1930-1934	0.473	0.019	25.120	<0.001	0.436	0.510
1935-1939	0.396	0.020	20.320	<0.001	0.358	0.434
1940-1944	0.317	0.021	15.450	<0.001	0.277	0.357
1945-1949	0.232	0.022	10.650	<0.001	0.189	0.275
1950-1954	0.139	0.023	5.960	<0.001	0.093	0.185
1955-1959	0.040	0.025	1.590	0.113	-0.009	0.089
1960-1964	-0.059	0.027	-2.190	0.028	-0.112	-0.006
1965-1969	-0.159	0.029	-5.520	<0.001	-0.216	-0.103
1970-1974	-0.275	0.031	-8.880	<0.001	-0.336	-0.215
1975-1979	-0.408	0.034	-12.170	<0.001	-0.474	-0.343
1980-1984	-0.556	0.037	-14.960	<0.001	-0.628	-0.483
1985-1989	-0.714	0.043	-16.620	<0.001	-0.799	-0.630
1990-1994	-0.883	0.056	-15.730	<0.001	-0.993	-0.773
1995-1999	-1.050	0.102	-10.340	<0.001	-1.249	-0.851
2000-2004	-1.223	0.377	-3.240	0.001	-1.963	-0.484
Intercept	6.992	0.020	355.560	<0.001	6.953	7.030
Log likelihood	-446.739					
AIC	10.772					
BIC	-138.071					

Supplementary Table 5. Age, period and cohort effects of gout prevalence in China (for women)

Factors	Effect coefficient	Standard error	Z value	p-value	95% CI	
					Lower	Upper
Age						
15-19	-3.844	0.216	-17.770	<0.001	-4.268	-3.420
20-24	-2.003	0.082	-24.470	<0.001	-2.164	-1.843
25-29	-1.046	0.056	-18.580	<0.001	-1.156	-0.935
30-34	-0.508	0.046	-10.950	<0.001	-0.599	-0.417
35-39	-0.146	0.040	-3.650	<0.001	-0.225	-0.068
40-44	0.099	0.035	2.800	<0.001	0.030	0.168
45-49	0.277	0.031	8.950	<0.001	0.216	0.338
50-54	0.432	0.027	16.070	<0.001	0.379	0.484
55-59	0.618	0.023	27.030	<0.001	0.574	0.663
60-64	0.796	0.019	41.100	<0.001	0.758	0.834
65-69	0.960	0.017	57.300	<0.001	0.927	0.992
70-74	1.077	0.016	69.450	<0.001	1.046	1.107
75-79	1.114	0.016	69.610	<0.001	1.082	1.145
80-84	1.107	0.018	61.500	<0.001	1.071	1.142
85-89	1.069	0.021	50.240	<0.001	1.027	1.110
Period						
1994	-0.427	0.015	-28.690	<0.001	-0.456	-0.397
1999	-0.375	0.011	-33.250	<0.001	-0.397	-0.353
2004	-0.056	0.008	-6.680	<0.001	-0.073	-0.040
2009	0.208	0.008	25.610	<0.001	0.192	0.224
2014	0.241	0.011	22.980	<0.001	0.221	0.262
2019	0.409	0.013	30.510	<0.001	0.383	0.436
Cohort						
1905-1909	1.014	0.041	24.980	<0.001	0.935	1.094
1910-1914	0.916	0.035	26.350	<0.001	0.848	0.984
1915-1919	0.815	0.031	26.080	<0.001	0.754	0.876
1920-1924	0.717	0.029	24.710	<0.001	0.660	0.774
1925-1929	0.630	0.028	22.580	<0.001	0.575	0.685
1930-1934	0.545	0.028	19.710	<0.001	0.491	0.599
1935-1939	0.455	0.029	15.770	<0.001	0.399	0.512
1940-1944	0.354	0.031	11.480	<0.001	0.294	0.415
1945-1949	0.243	0.034	7.260	<0.001	0.178	0.309
1950-1954	0.122	0.037	3.330	0.001	0.050	0.194
1955-1959	-0.002	0.040	-0.060	0.956	-0.081	0.077
1960-1964	-0.121	0.044	-2.760	0.006	-0.208	-0.035
1965-1969	-0.239	0.048	-5.000	<0.001	-0.333	-0.145
1970-1974	-0.367	0.052	-7.020	<0.001	-0.469	-0.264
1975-1979	-0.498	0.057	-8.690	<0.001	-0.610	-0.386
1980-1984	-0.631	0.064	-9.800	<0.001	-0.758	-0.505
1985-1989	-0.768	0.076	-10.090	<0.001	-0.917	-0.619
1990-1994	-0.911	0.101	-9.000	<0.001	-1.109	-0.712
1995-1999	-1.061	0.174	-6.090	<0.001	-1.402	-0.720
2000-2004	-1.214	0.550	-2.200	0.027	-2.293	-0.135
Intercept	5.892	0.029	200.210	<0.001	5.834	5.950
Log likelihood	-394.335					
AIC	9.607					
BIC	-143.458					

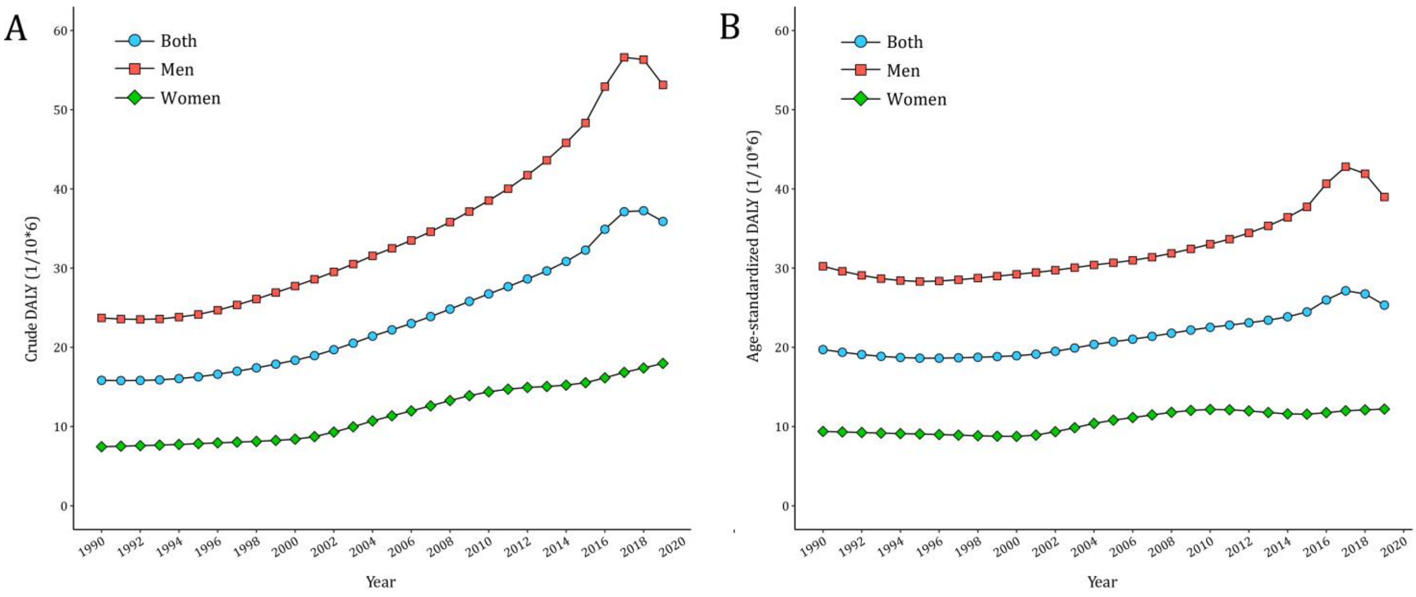

Supplementary Figure 1. Trends of gout prevalence in China during 1990-2019
(A. crude DALY of gout; B. age-standardized DALY of gout)

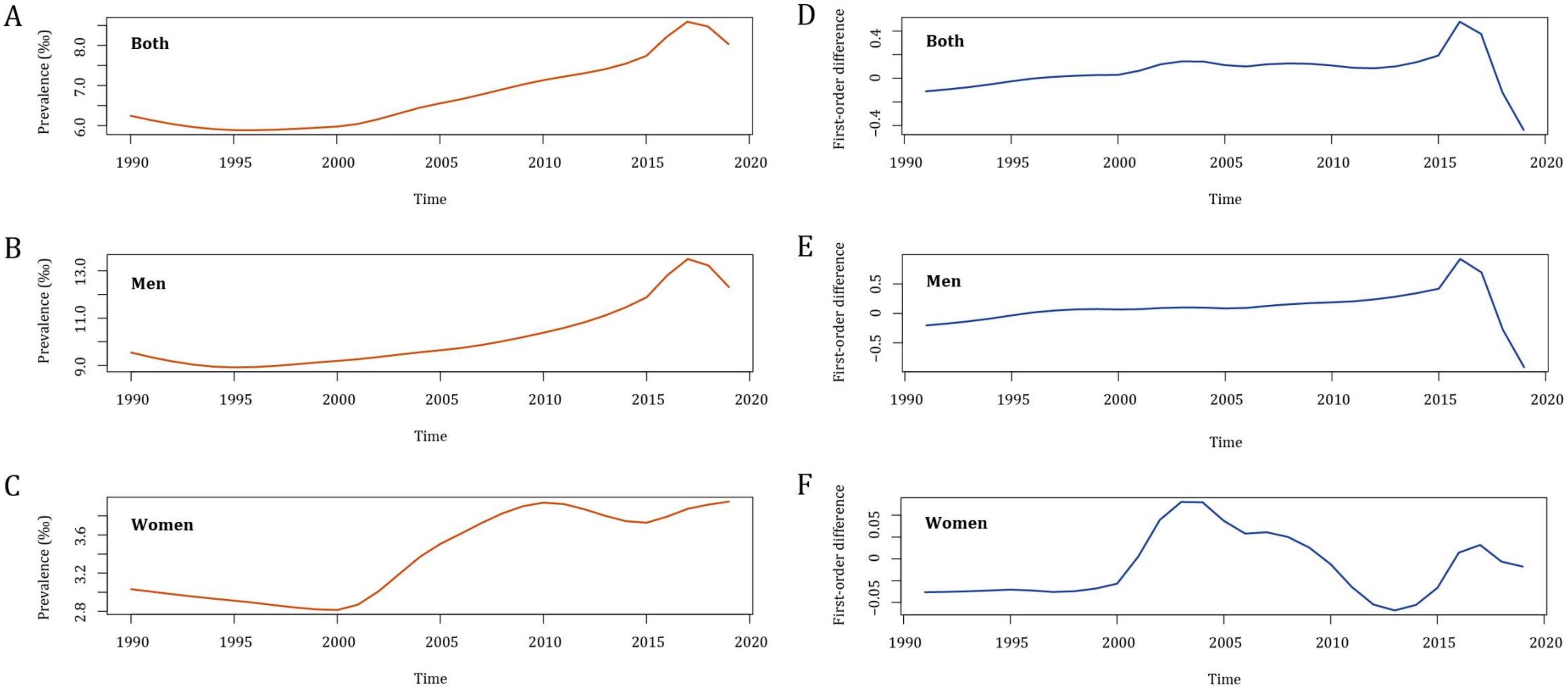

Supplementary Figure 2. Timing diagram of gout prevalence (A-C: Age-standardized prevalence of gout; D-F: Age-standardized prevalence of gout after first-order differencing)

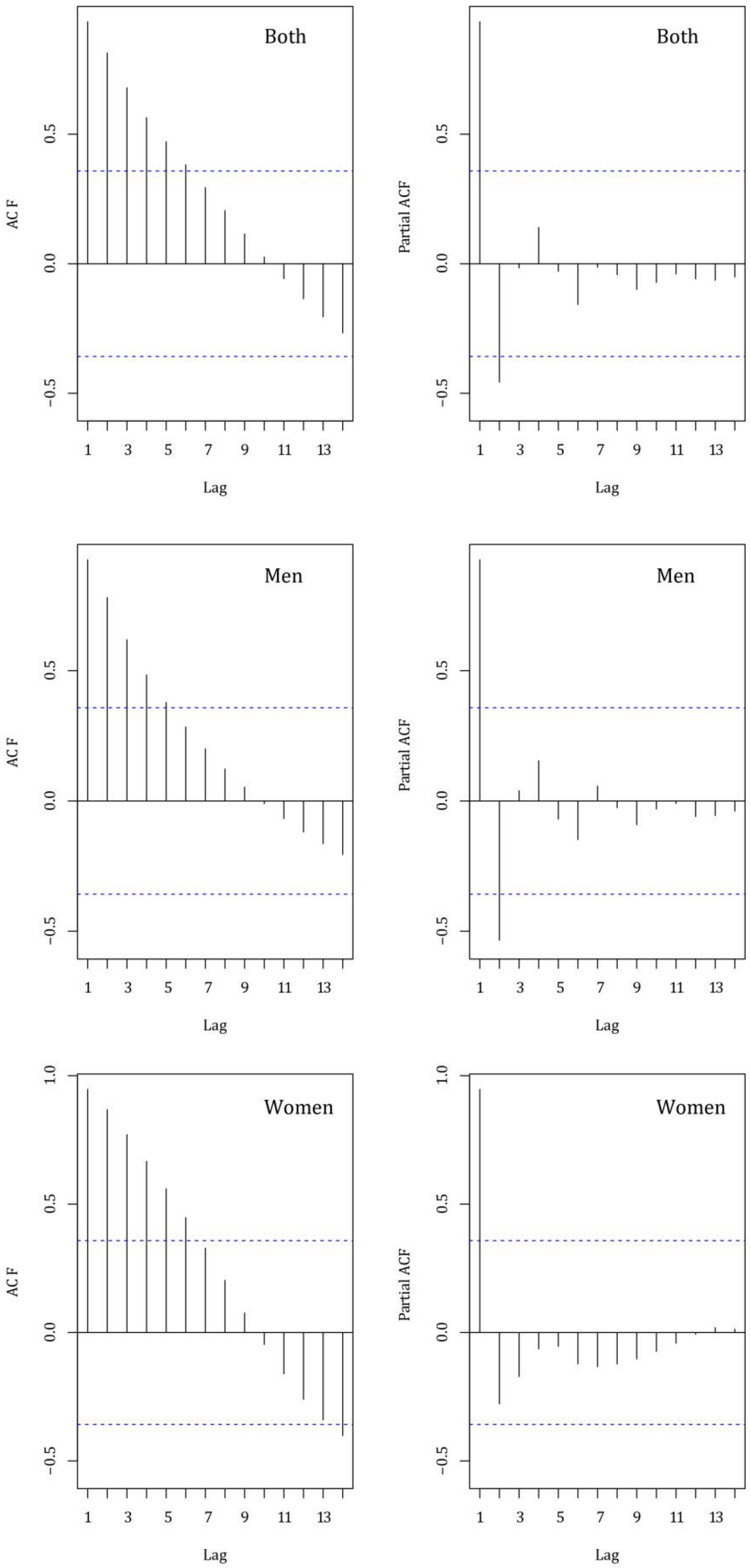

Supplementary Figure 3. Autocorrelation function and partial autocorrelation function graphs of original data

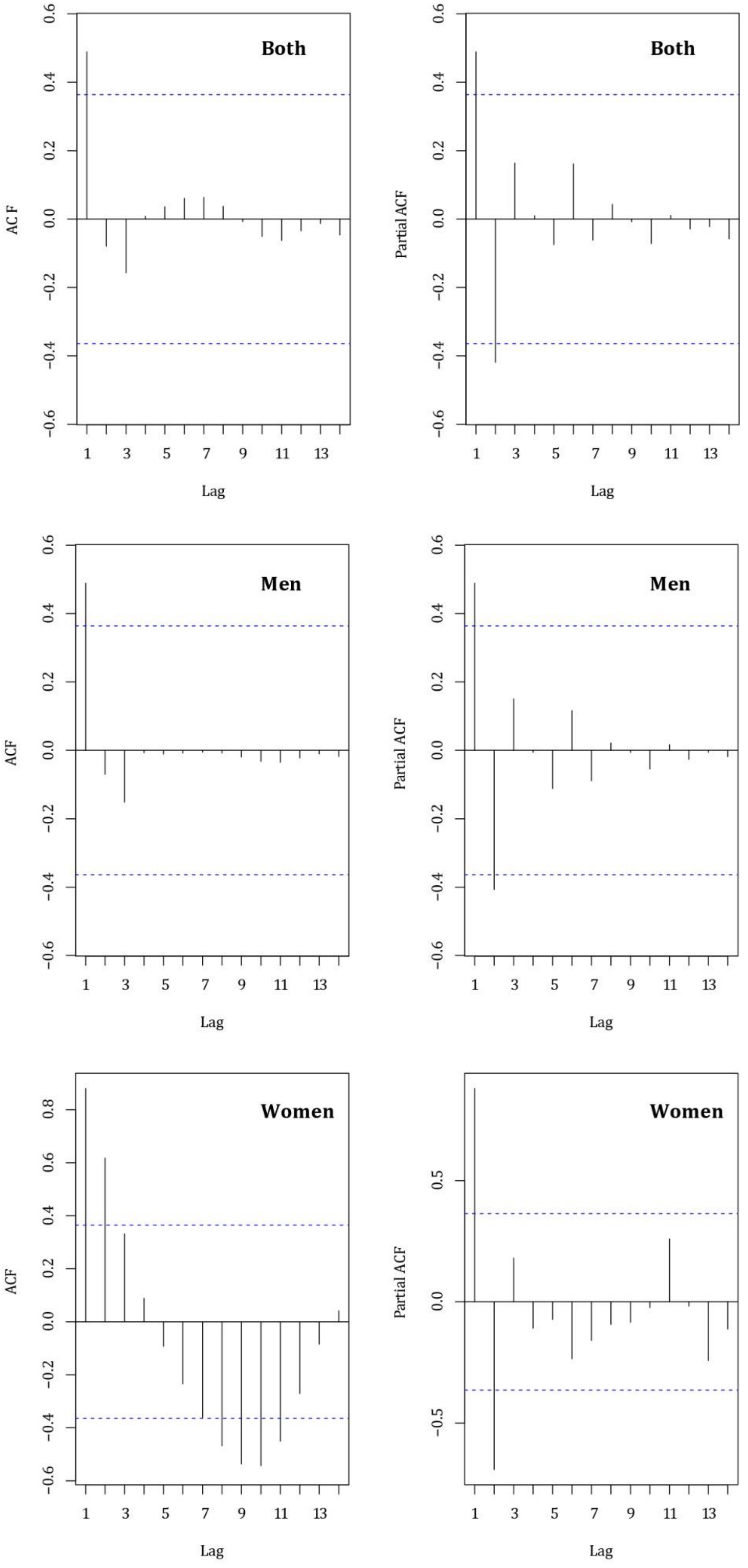

Supplementary Figure 4. Autocorrelation function and partial autocorrelation function graphs after one order differencing

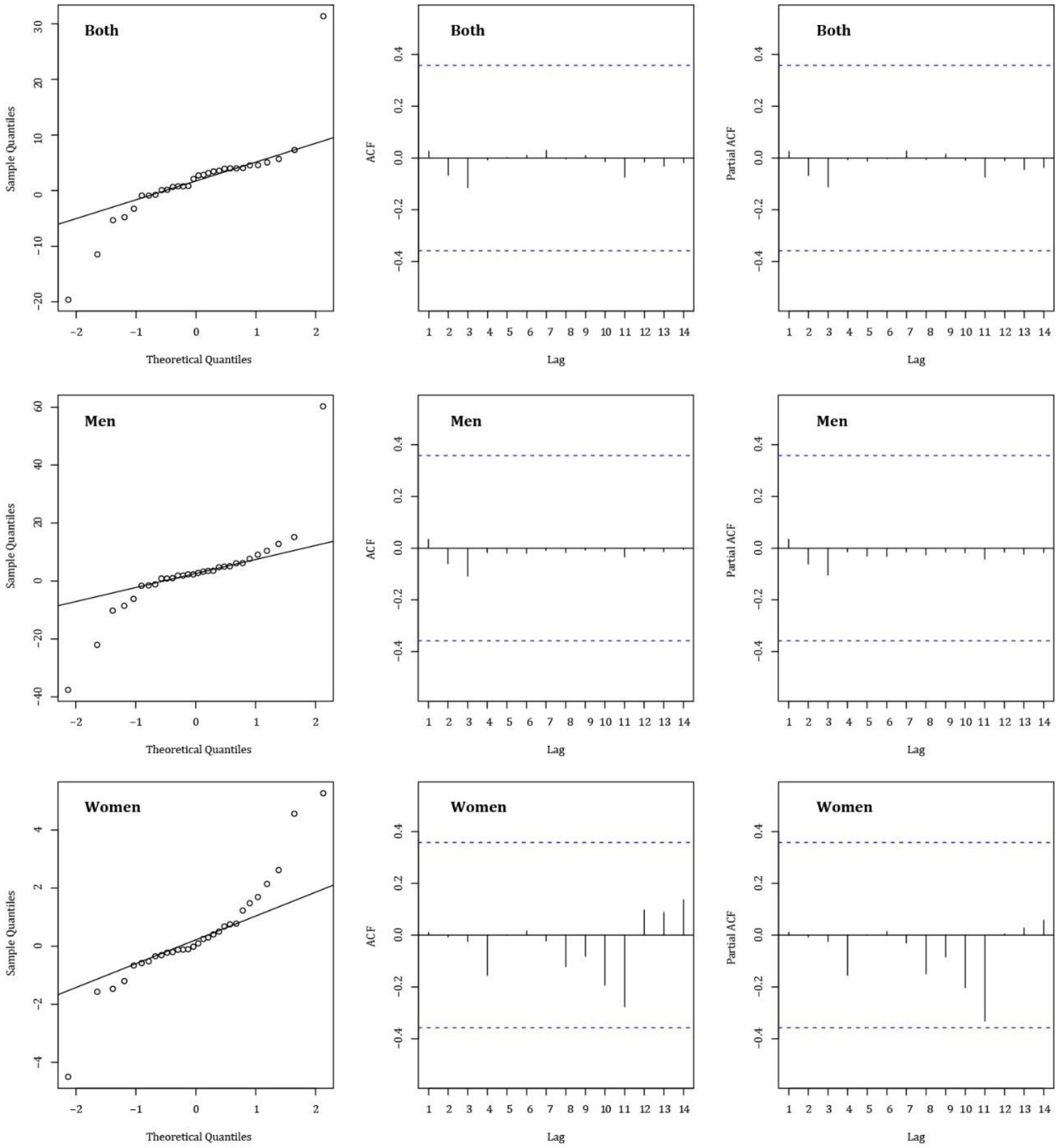

Supplementary Figure 5. Residual Q-Q plots, autocorrelation function and partial autocorrelation graphs of ARIMA models

Supplementary Table 6. The white noise test for differential time series and ARIMA models

	Differencing		ARIMA models	
	Ljung-Box λ^2	p-value	Ljung-Box λ^2	p-value
Both	1.620	0.203	0.023	0.881
Men	1.594	0.207	0.040	0.842
Women	2.607	0.106	0.004	0.949

Supplementary Table 7. The predictive capacity of ARIMA models

	ME	RMSE	MAE	MPE	MAPE	MASE
Both	1.619	7.835	4.757	0.238	0.643	0.380
Men	2.582	14.843	8.512	0.250	0.741	0.385
Women	0.361	1.794	1.157	0.116	0.335	0.189

ME: Mean Error, RMSE: Root Mean Squared Error, MAE: Mean Absolute Error, MPE: Mean Percentage Error, MAPE: Mean Absolute Percentage Error, MASE: Mean Absolute Scaled Error

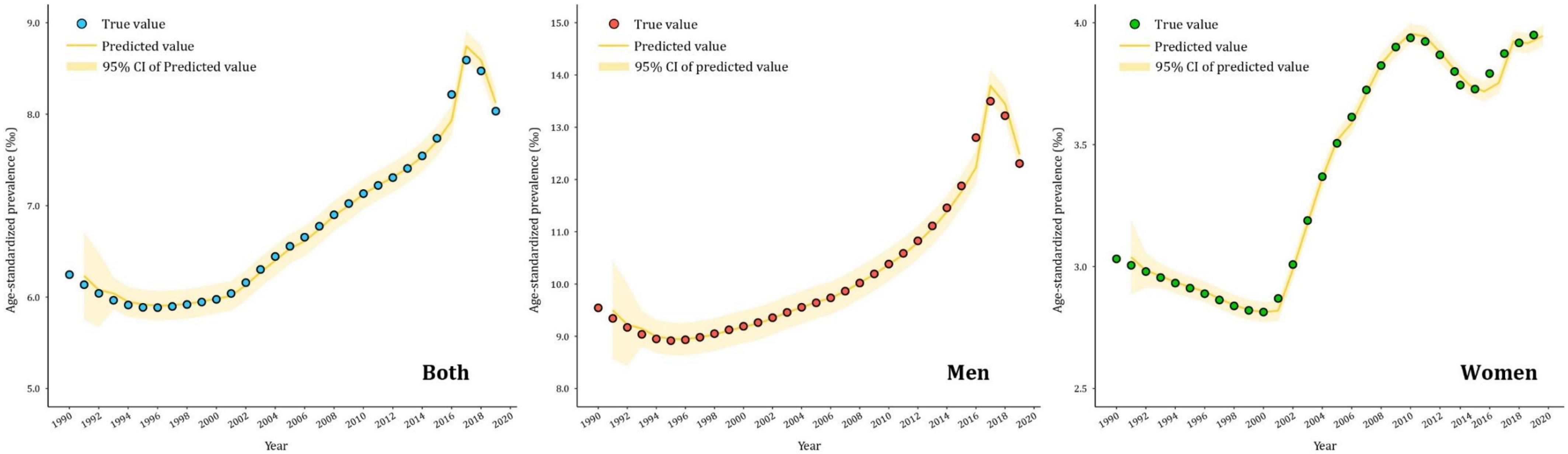

Supplementary Figure 6. Comparison of the true and predicted values of age-standardized prevalence of gout in ARIMA models (the blue, red and green dot lines represent the true trend of age-standardized prevalence of gout; the yellow lines represent the predicted trend and the light-yellow shaded regions represent the 95% confidence interval of predicted values)

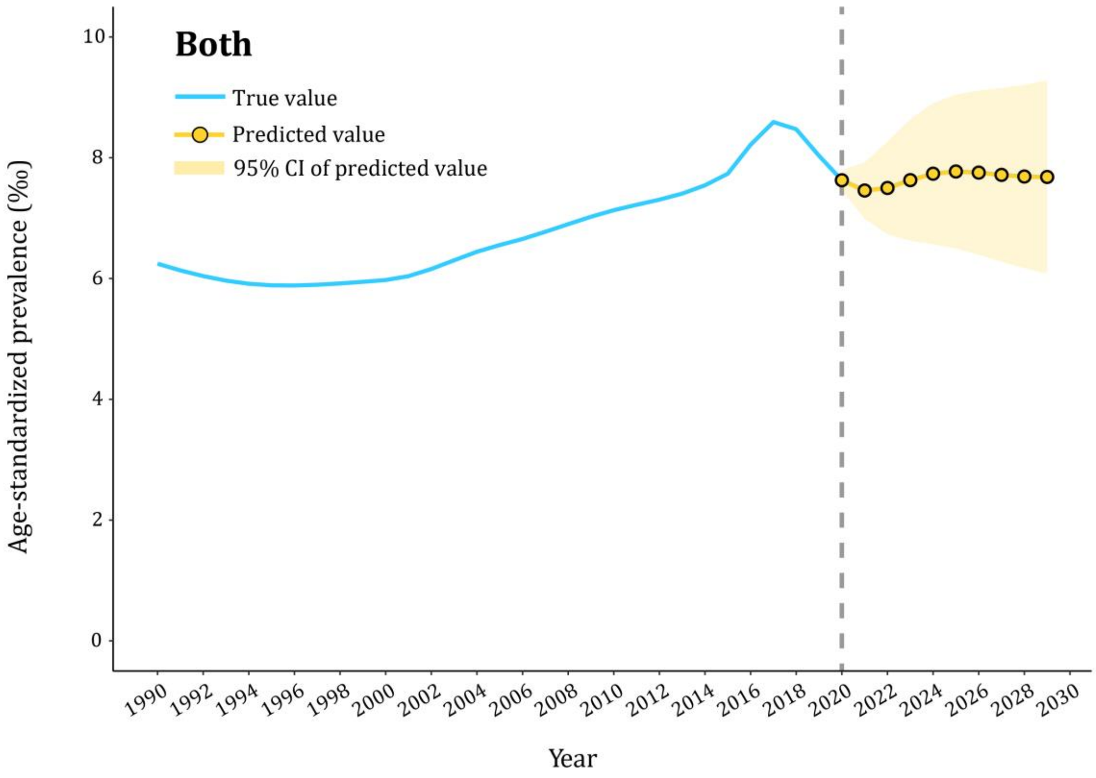

Supplementary Figure 7. Predicted trends of gout prevalence in China over ten years (2020~2029) (the yellow lines represent the predicted trend and the light-yellow shaded regions represent the 95% confidence interval of predicted values; the gray dot vertical line split data into true value (1990-2019) and predicted value (2020-2029))
